# Characteristics and Their Clinical Relevance of Respiratory Syncytial Virus Types and Genotypes Circulating in Northern Italy in Five Consecutive Winter Seasons

**DOI:** 10.1371/journal.pone.0129369

**Published:** 2015-06-05

**Authors:** Susanna Esposito, Antonio Piralla, Alberto Zampiero, Sonia Bianchini, Giada Di Pietro, Alessia Scala, Raffaella Pinzani, Emilio Fossali, Fausto Baldanti, Nicola Principi

**Affiliations:** 1 Pediatric Highly Intensive Care Unit, Department of Pathophysiology and Transplantation, Università degli Studi di Milano, Fondazione IRCCS Ca’ Granda Ospedale Maggiore Policlinico, Milan, Italy; 2 Molecular Virology Unit, Microbiology and Virology Department Fondazione IRCCS Policlinico San Matteo, Pavia, Italy; 3 Emergency Unit, Fondazione IRCCS Ca’ Granda Ospedale Maggiore Policlinico, Milan, Italy; 4 Section of Microbiology, Department of Clinical, Surgical, Diagnostic and Pediatric Sciences, University of Pavia, Pavia, Italy; Imperial College London, UNITED KINGDOM

## Abstract

In order to investigate the genetic diversity and patterns of the co-circulating genotypes of respiratory syncytial virus (RSV) and their possible relationships with the severity of RSV infection, we studied all of the RSV-positive nasopharyngeal samples collected from children during five consecutive winters (2009–2010, 2010–2011, 2011–2012, 2012–2013 and 2013–2014). The RSVs were detected using the respiratory virus panel fast assay and single-tube RT-PCR, their nucleotides were sequenced, and they were tested for positive selection. Of the 165 positive samples, 131 (79.4%) carried RSV-A and 34 (20.6%) RSV-B; both groups co-circulated in all of the study periods, with RSV-A predominating in all the seasons except for winter 2010–2011, which had a predominance of RSV-B. Phylogenetic analysis of the RSV-A sequences identified genotypes NA1 and ON1, the second replacing the first during the last two years of the study period. The RSV-B belonged to genotypes BA9 and BA10. BA9 was detected in all the years of the study whereas BA only desultorily. Comparison of the subjects infected by RSV-A and RSV-B types did not reveal any significant differences, but the children infected by genotype A/NA1 more frequently had lower respiratory tract infections (p<0.0001) and required hospitalisation (p = 0.007) more often than those infected by genotype A/ON1. These findings show that RSV has complex patterns of circulation characterised by the periodical replacement of the predominant genotypes, and indicate that the circulation and pathogenic role of the different RSV strains should be investigated as each may have a different impact on the host. A knowledge of the correlations between types, genotypes and disease severity may also be important in order to be able to include the more virulent strains in future vaccines.

## Introduction

Human respiratory syncytial virus (RSV) is the second most frequent cause of respiratory infections, and the main cause of severe cases of bronchiolitis and pneumonia in infants and young children [1].

Classified in the *Pneumovirus* genus of the family *Paramyxoviridae*, it is an enveloped virus with a negative-sense single-strained RNA genome that encodes for 11 proteins [2], including two surface glycoproteins that are antigenically significant because they induce neutralising antibody responses: fusion protein (F protein) and attachment glycoprotein (G protein). F protein is conserved, whereas the C-terminal third hypervariable region of the G protein is subject to rapid mutation [2]. On the basis of their reactions with monoclonal antibodies, the viral strains have been separated into two major serotypes (RSV-A and RSV-B), and nucleotide sequence analysis has led to the identification of 11 RSV-A (GA1-GA7, SAA1, NA1-NA2, and ON1) [2–4] and 23 RSV-B genotypes, (GB1-GB4, SAB1-SAB3, SAB4, URU1, URU2, BA1-BA12 and THB) [5–13]. Multiple genotypes can co-circulate during successive epidemic seasons, but a new genotype (or one spreading from another country) may replace previously dominant strains [5,14,15].

The rapid spread of a novel RSV-A genotype (A/ON1, replacing the ancestor A/NA1) has recently been documented in a number of countries [13,16–21]. RSV molecular epidemiological surveillance is essential for detecting the emergence of new viral strains, identifying or predicting their clinical relevance, selecting candidate strains for vaccine development, and preparing public health intervention strategies based on phylogeographic analysis.

The clinical manifestations of RSV infection can range from mild upper respiratory tract illness (URTIs) or acute otitis media to severe and potentially life-threatening lower respiratory tract involvement (LRTIs) [22]. It has been demonstrated that various host characteristics and a number of factors, including viral types and genotypes, can influence the clinical course of RSV infection in children [1,22,23], but the real importance of each of them are still unclear. The main aim of this study was to investigate the genetic diversity and patterns of the co-circulating genotypes of RSV-A and RSV-B strains in Milan, and the possible associations between individual RSV genotypes and the clinical course of RSV infection.

## Methods

### Study design

In order to evaluate the circulation of the different RSV types and genotypes, and the possible relationships between their genetic characteristics and the severity of RSV infection, we studied all of the nasopharyngeal samples exclusively positive for RSV collected from children aged <2 years attending the Emergency Room of the University of Milan’s Fondazione IRCCS Ca’ Granda Ospedale Maggiore Policlinico because of influenza-like illness arising between November 1 and March 31 of five consecutive winters (2009–2010, 2010–2011, 2011–2012, 2012–2013 and 2013–2014). The samples were selected from those previously collected during a study specifically designed to analyse the impact of respiratory viruses in children aged <15 years with an influenza-like illness (as defined by the Italian Ministry of Health, http//www.ministerosalute.it) and without any underlying severe chronic disease [24]. In accordance with the Italian Ministry of Health, an ILI was defined as an acute respiratory disease of sudden onset, with fever (an axillary temperature of >38°C) and accompanied by at least one of the general symptoms of headache, generalized malaise, a feverish sensation (sweating and chills) or asthenia, and at one of the respiratory symptoms of cough, pharyngodynia or nasal congestion.

Upon enrolment, the patients’ demographic characteristics and medical history were systematically recorded using standardised written questionnaires [24] and, after a complete clinical examination, they were classified into disease groups (acute otitis media, rhinosinusitis, pharyngitis, croup, infectious wheezing, acute bronchitis, pneumonia, diarrhea and gastroenteritis) on the basis of signs and/or symptoms using well-established criteria [24].

A nasopharyngeal sample was collected from all of the children using a pernasal flocked swab, which was stored in a tube containing 1 mL of universal transport medium (Copan Italia, Brescia, Italy).

The children’s medical history was re-evaluated by means of follow-up interviews and a clinical examination by trained investigators using standardized questionnaires [24] 5–7 days after enrolment, and then every two days until the resolution of their illness. All of the data of the study children were verified against their medical records.

Both the original study and this extension were approved by the Ethics Committee of the Fondazione IRCCS Ca’ Granda Ospedale Maggiore Policlinico, and written informed consent obtained from a parent or legal guardian of the enrolled children (those aged >8 years were asked to give their assent).

### Identification of respiratory viruses

Viral nucleic acids were extracted from the samples by means of a NucliSENS EasyMAG automated extraction system (BioMéerieux, Craponne, France), and the extract was tested for respiratory viruses using the respiratory virus panel xTAG RVP FAST v2 (Luminex Molecular Diagnostics, Inc., Totonto, Canada), which simultaneously detects influenza A virus (subtypes H1 or H3), influenza B virus, respiratory syncytial virus, parainfluenzavirus-1, parainfluenzavirus-2, parainfluenzavirus-3 and parainfluenzavirus-4, adenovirus, human metapneumovirus, coronaviruses 229E, NL63, OC43 and HKU1, enterovirus/rhinovirus and human bocavirus. The samples that were positive for RSV and negative for other viruses were stored at -80°C.

### RSV types and genotypes

The RSV-positive viral nucleic acid extracts were re-tested for confirmation using a single-tube RT-PCR kit (TaqMan One-Step RT-PCR Master Mix Reagents Kit, Applied Biosystems, New Jersey, USA) and a 7,900HT real-time PCR system (Applied Biosystems, New Jersey, USA). The N genes of RSV-A and RSV-B were targeted for the confirmation with primers and probes with minor modifications, according to Van Elden *et al*. [25]. Each tube contained a 25 μL reaction mix that included 2.5 μL isolated RNA, 0.9 μM forward primer, 0.9 μM reverse primer and 0.25 μM probe. The primers and probes for the TaqMan amplification of viral RNA are described in Table 1.

**Table 1:** Primers and probes for the TaqMan real-time PCR used to detect viral RNA from RSV-A and B.

RSV type (target gene)	Primer and probe	Sequence
A (N gene)	RSA-1	5’-AGATCAACTTCTGTCATCCAGCAA-3’
	RSA-Rev	5’-TGTGTTTCTGCACATCATAATTAGGA-3’
	RSA-probe	FAM-ACACCATCCAACGGAGCACAGGAGA-TAMRA
B (N gene)	RSB-1	5’-AAGATGCAAATCATAAATTCACAGGA-3’
	RSB-Rev	5’-TGATATCCAGCATCTTTAAGTATCTTTATAGTG-3’
	RSB probe	FAM-AGGTATGTTATATGCTATGTCCAGGTTAGGAAGGGAA-TAMRA

RSV cDNA was obtained from the extract using MultiScribe reverse transcriptase and random hexamers (TaqMan reverse transcription reagents, Applied Biosystems International, New Jersey, USA). The second hypervariable region of the G protein of the RSV-positive samples was the target for the genotyping using the forward primer, ABG490 (ATGATTWYCAYTTTGAAGTGTTC), that corresponds to bases 497–519 of the G protein gene of strain A2 (group A prototype strain, GenBank Acc. No. M11486) and BA4128/99B (group BA prototype strain, GenBank Acc. No. AY333364) and the reverse primer, F164 (GTTATGACACTGGTATACCAACC), that corresponds to bases 164–186 (with one mismatch) of the F protein gene of strain A2 and bases 164–186 in sequences of strain 18537 (a group B strain, GenBank Acc. No. D00334) [26, 27].

Total cDNA was used in a PCR under the following conditions: 94°C for 3 min, 40 cycles of 94°C for 35 sec, 50°C for 45 sec, and 72°C for 1 min, followed by final extension at 72°C for 10 min. The reaction mixture contained 1x PCR buffer (GE Healthcare, Uppsala, Sweden), 10 mM dNTP, 0.25 mM MgCl2, 0.25 mM of ABG490 and F164 primers, and 5 U of Taq polymerase (GE Healthcare, Uppsala, Sweden). The amplified products of 607/610 bp (group A/B viruses) and 670 bp (BA viruses) were analysed by means of electrophoresis on a 1.5% agarose gel and then purified using a QIAquick Gel Extraction Kit (QIAGEN, Venlo, Netherlands). The sequencing reactions were set up using purified DNA, one of the specific primers used in the PCR, and the BigDye Terminator v3.1 Cycle Sequencing Kit (Applied Biosystems International, New Jersey, USA) in accordance with the protocol recommended by the manufacturer. The sequencing and sequence analyses were performed on a 3130 Genetic Analyser (Applied Biosystems International, New Jersey, USA). The nucleotide sequence spanned bases 5,274–5,543 (270 nt) of prototype strain A2; in the case of group B viruses, the sequence corresponded to bases 652–981 (330 nt) of BA4128/99B, a prototype genotype B strain. All of the alignments were made using ClustalX 2.1 and BioEdit (version 7.1.3.0) software (Ibis Biosciences, Carlsbad, CA).

Phylogenetic trees of the G protein gene were generated using the neighbour-joining method, p-distance model with Molecular Evolutionary Genetics Analyses (MEGA) software, version 5.05 [28]. Bootstrap probabilities for 1,000 iterations were calculated to evaluate confidence estimates. The graphs were made using GraphPad Prism version 5.01 for Windows (GraphPAD Software, San Diego, CA). The nucleotide sequences identified in this study have been submitted to GenBank database with accession numbers KP284582-KP284746.

### Selective pressure

Tests for positive selection were conducted using single-likelihood ancestor counting (SLAC), fixed-effects likelihood (FEL), random effects likelihood (REL), internal branch fixed-effects likelihood (IFEL), the mixed effects model of evolution (MEME), and fast unconstrained Bayesian approximation (FUBAR) on the Datamonkey server [29], with the *dN/dS* ratios being calculated using the SLAC and FEL codon-based maximum likelihood approaches. SLAC counts the number of non-synonymous changes per non-synonymous site (*dN*) and tests whether it is significantly different from the number of synonymous changes per synonymous site (*dS*). FEL estimates the ratios of non-synonymous to synonymous changes for each site in an alignment [30]. The IFEL method is similar to FEL, but tests for site-by-site selection only along internal branches of the phylogeny. In order to avoid an excessive false-positive rate, sites with SLAC, FEL, IFEL and MEME *p*-values of <0.1 and a FUBAR posterior probability of >0.90 were accepted as candidates for selection.

### Statistical analysis

Descriptive statistics of the responses were generated. The continuous variables are given as mean values ± standard deviation (SD), and the data were analysed using a two-sided Student’s t test after checking data were normally distributed (based on the Shapiro-Wilk statistic) or a two-sided Wilcoxon’s rank-sum test otherwise; the categorical variables are given as numbers and percentages, and the between-group data were compared using contingency table analysis with the χ^2^ or Fisher’s exact test, as appropriate. All of the analyses were two tailed, and made using SAS version 9.2 software (Cary, NC, USA); *p-*values of ≤0.05 were considered statistically significant.

## Results

### Prevalent RSV types and genotypes

Of the 165 exclusively RSV-positive nasopharyngeal samples screened using the respiratory virus panel and re-confirmed by Taqman Real-Time PCR 131 (79.4%) carried RSV-A and 34 (20.6%) RSV-B. Both groups co-circulated in all of the study periods, with RSV-A predominating in all the seasons except for 2010–2011, which had a predominance of RSV-B. The distribution of the RSV strains is shown in Table 2: 29 cases (25 RSV-A, four RSV-B) in 2009–2010; 13 cases (three RSV-A, 10 RSV-B) in 2010–2011; 36 cases (35 RSV-A, one RSV-B) in 2011–2012; 30 cases (22 RSV-A, eight RSV-B) in 2012–2013; and 57 cases (46 RSV-A, 11 RSV-B) in 2013–2014. No case of co-infection with the two groups was identified.

**Table 2:** RSV types and genotypes detected in the studied nasopharyngeal samples.

	No. (%) of samples with the indicated species and genotype					
Type and genotype	Total	09–10	10–11	11–12	12–13	13–14
RSV-A / NA1	62 (37.6)	25 (40.3)	2 (3.2)	35 (56.5)	0	0
RSV-A / ON1 –group A	29 (17.6)	0	1 (3.5)	0	17 (58.6)	11 (37.9)
RSV-A / ON1 –group A1	16 (9.7)	0	0	0	0	16 (100.0)
RSV-A / ON1 –group B	24 (14.5)	0	0	0	5 (20.8)	19 (79.2)
RSV-B / BA-9	26 (15.8)	4 (15.4)	4 (15.4)	1 (3.8)	6 (23.1)	11 (42.3)
RSV-B / BA-10	8 (4.8)	0	6 (75.0)	0	2 (25.0)	0
**Total RSV**	**165**	**29**	**13**	**36**	**30**	**57**

p<0.001 (chi-squared test for independence between autumn-winter season and species/genotype)

### Phylogenetic analyses and amino acid signatures

**Fig 1:**
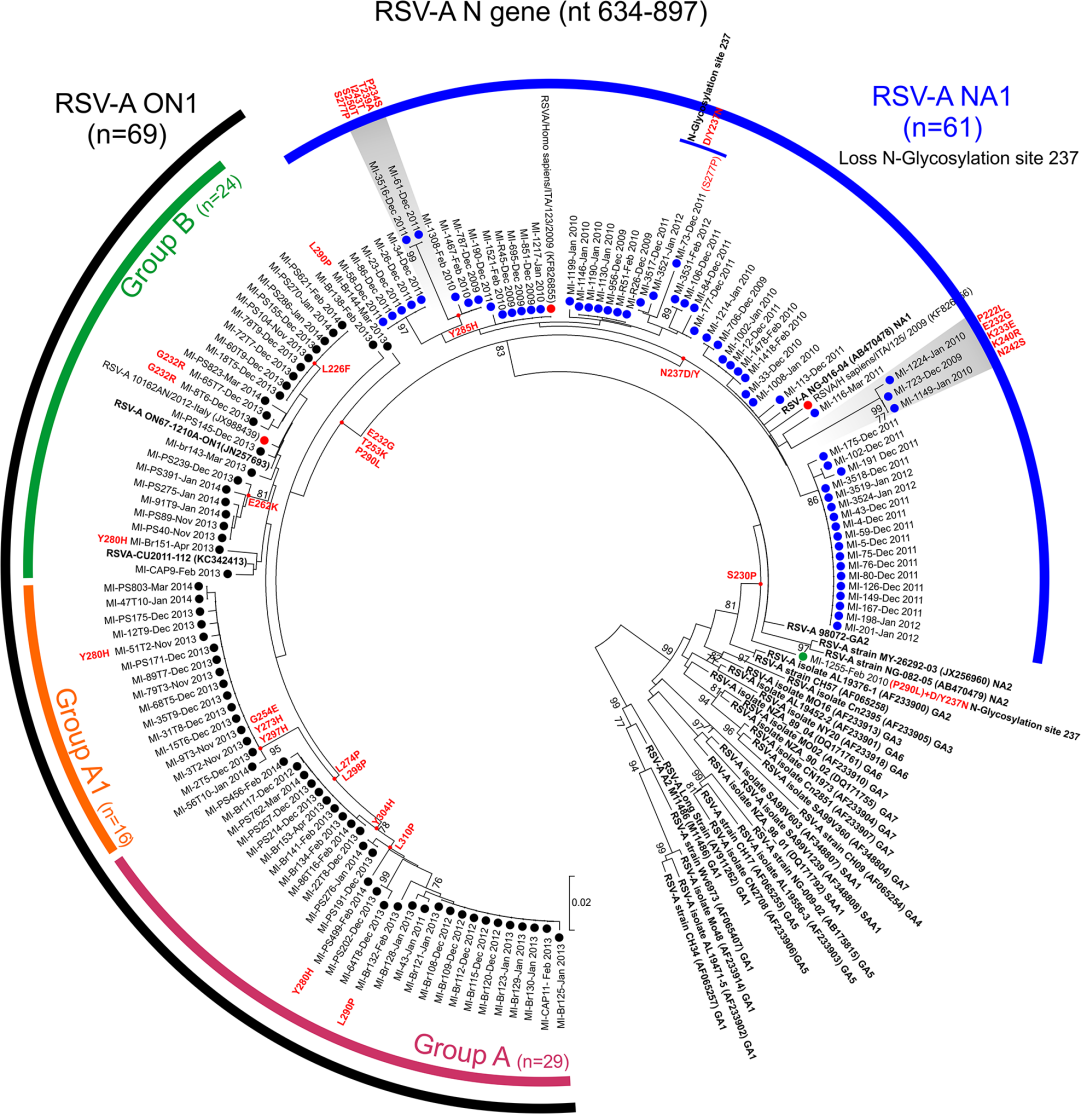
Phylogenetic tree based on partial G gene sequences of RSV-A strains (nt 634–897). The sequences originally found in this study are indicated by black, blue and green circles (n = 131); the other Italian reference sequences are indicated by red circles (n = 3). The RSV-A reference stains are in bold (n = 32). Significant amino acid changes are reported along the tree branch or near the strain name. The percentage of replicate trees in which the associated taxa clustered together in the bootstrap test (1000 replicates) are shown next to the branches. The evolutionary distances were computed using the Tamura-Nei method.

Phylogenetic analysis of the RSV-A sequences led to the identification of 61/131 samples (46.5%) carrying genotype NA1 (blue circles in Fig 1) and 69/131 (52.7%) carrying genotype ON1 strains (black circles in Fig 1) and 1/131 (0.8%) with undetermined genotype (green circle in Fig 1). The p-distances within the NA1 and ON1 genotypes were respectively 2.4 ± 0.3% and 2.3 ± 0.5%, and the p-distance between NA1 and ON1 was 5.2 ± 1.2%.

The NA1 strain sequences were characterised by N237D/Y amino acid substitutions, a change involved in the loss of a potential glycosylation site in position 237. Only three sequences (MI-3521-Jan2011, mi-3517-Dec2011, and Mi-73-Dec2011) were observed with a recovered N-glycosylation site in positon 237. It is worth noting that two divergent clusters (highlighted in grey in Fig 1) were identified with several amino acid changes: one characterised by amino acid changes P222L, E232G, K233E, K240R and N242S, and the other by amino acid changes P234S, T239A, I243T, S250T and S277P. RSV-A NA1 was the only genotype identified in the samples collected during the winter seasons between 2009–2010 to 2011–2012, but was no longer present in the later samples.

A single swab was positive for genotype ON1 in 2011–2012, but this genotype subsequently completely replaced NA1. The RSV-A strains belonging to the ON1 genotype were characterised by amino acid changes E232G, T253K and P290L (RSV-A NA1 numbering). Within the ON1 genotype, three different phylogenetic subgroups (group A: n = 29 strains; group A1; n = 16 strains; and group B: n = 24 strains), could be distinguished in the phylogenetic tree shown in Fig 1 on the basis of some amino acid substitutions in comparison with the first-described ON1 strain. The sequences belonging to group A were characterised by amino acid changes L274P, L298P and Y304H, and those in group A1 by the amino acid changes G254E, Y273H and Y297H; no unique amino acid changes were observed in the sequences belonging to group B.

**Fig 2:**
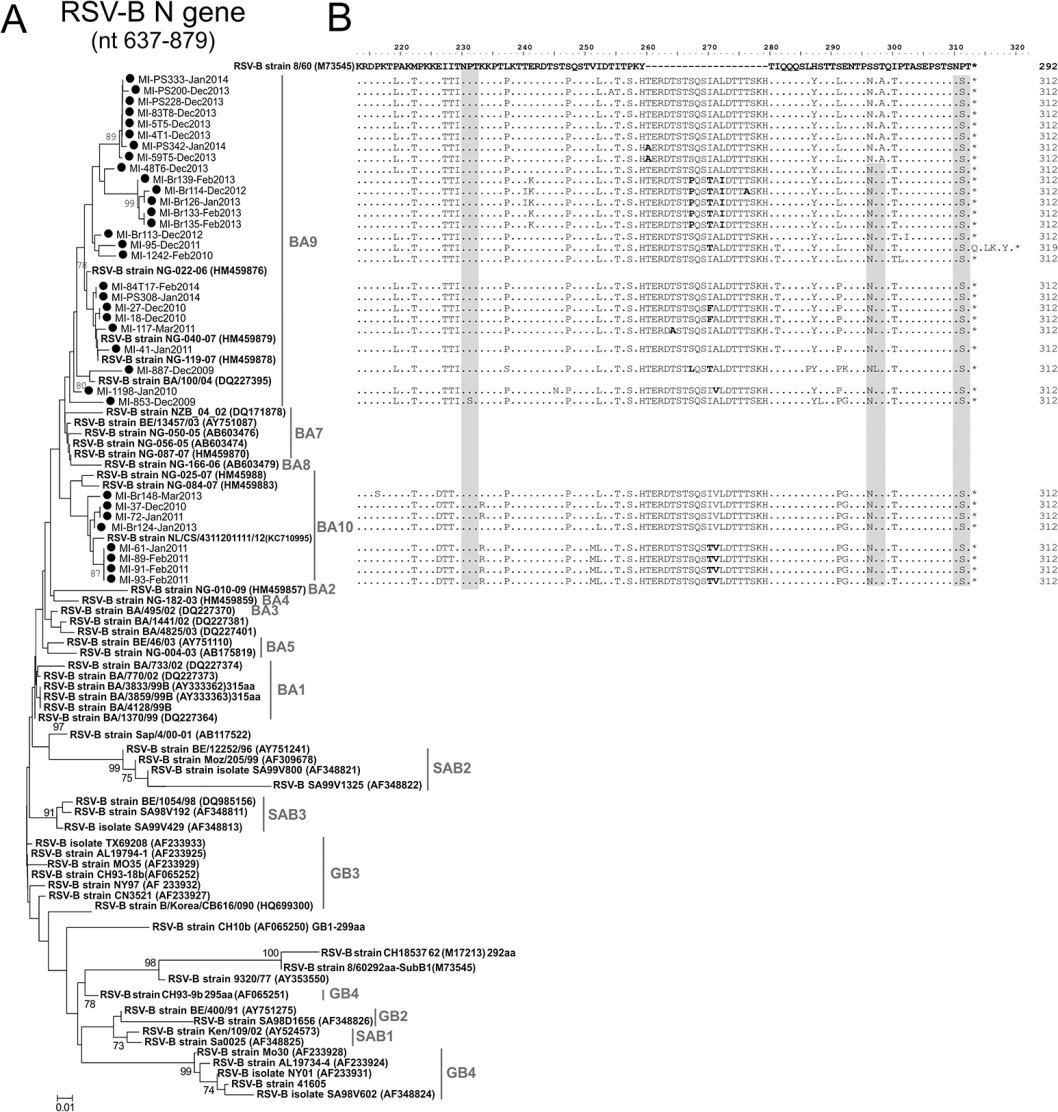
(A) Phylogenetic tree based on partial G gene sequences of RSV-B strains (nt 637–897). The sequences originally found in this study are indicated by black circles (n = 34). The RSV-B reference stains are in bold (n = 55). The percentage of replicate trees in which the associated taxa clustered together in the bootstrap test (1000 replicates) are shown next to the branches. (B) Alignment of deduced G protein amino acid sequence of RSV-B strains. The gaps are indicated by dashes (-) and the conserved amino acid residues by dots (.). The length of the G gene is shown at the end of the sequence of each strain. The amino acids included in glycosylation sites are highlighted by grey boxes.

Phylogenetic analysis of the samples positive for RSV-B showed that 26/34 (76.5%) belonged to genotype BA9 and 8/34 (23.5%) to genotype BA10 (Fig 2A). The first was detected during all of the studied seasons, whereas BA10 was detected only in the 2010–2011 and 2012–2013 seasons. All of the RSV-B strains identified in this study carried the 60 nt duplication in the second hypervariable region of the G protein (Fig 2B): all but one strain had a predicted G protein length of 312 amino acids, whereas strain MI-95-Dec2011 had a G protein length of 319 amino acids. In addition, a number of non-synonymous amino acid substitutions were observed in the duplicated region (indicated in bold in Fig 2B). The p-distances in genotypes GB9 and GB10 were respectively 3.5 ± 0.7% and 1.0 ± 0.4%, whereas the p-distance between GB9 and GB10 was 7.4 ± 1.7%. The nucleotide sequences identified in this study have submitted to GenBank database with accession numbers KP284582-KP284746.

### Analyses of selective pressure

A global analysis of selective pressure made using the SLAC model indicated an estimated overall *dN/dS* ratio of 0.81 for RSV-A and 0.67 for RSV-B. Overall, the site-specific analyses identified two sites (274, 310) in the RSV-A and one (270) in the RSV-B alignment as being under positive selection by at least three of the methods used (SLAC, FEL, REL, FUBAR and MEME). Site 270 was included in the 60 nt duplication region of the RSV-B strains. A number of negatively selected sites were identified in both the RSV-A and RVS-B G protein alignment by means of different methods (Table 3).

**Table 3:** Positive and negative selected sites for RSV-A and RSV-B strains identified in this study.

Methods	RSV-A codon		RSV-B codon	
	Positive	Negative	Positive	Negative
SLAC	274, 298	227, 294	None	216, 285
FEL	274, 310	223, 227, 256, 266	270	216, 223, 249, 269, 285, 295, 304
REL	262, 274,	none	270	214, 216, 220, 223, 229, 238, 246, 249, 250, 253, 256, 257, 264, 269, 272, 285, 295, 299, 304, 307, 311, 312
FUBAR	262, 274	227, 239, 256, 266	270, 292	216, 223, 249, 269, 285, 295, 304
MEME	244, 274	none	270, 297	None
IFEL	237, 274, 298, 310	239, 280	219	216, 285

FEL: fixed-effects likelihood; FUBAR: fast unconstrained Bayesian approximation; IFEL: random effects likelihood; MEME: mixed effects model of evolution; REL: internal branch fixed-effects likelihood; SLAC: single-likelihood ancestor.

The IFEL model used to determine the selection pressure acting on the codons along the internal branches of the tree identified four positively selected codons (237, 274, 298, and 310) in the RSV-A sequences. This reflects the amino acid changes observed in the sequences of genotype NA1 (Fig 1), particularly the amino acid signatures representative of the sequence evolution of clusters A and A1 (L274P, L298P and L310P).

### Association between RSV types/genotypes and disease severity

Table 4 shows the demographic, clinical and laboratory data relating to the infected children. There were no statistically significant differences in any of the studied variables between those infected by RSV-A and those infected by RSV-B. Age and clinical manifestations of RSV infection were similar in the two groups, as were the hospitalisation rates, the duration of hospital stays, and all of the laboratory variables except for the white blood cell count, which was higher in the children with RSV-A infection.

**Table 4:** Comparison of individual RSV infections by demographic, clinical and laboratory variables.

Characteristic	RSV-A n = 131	RSV-B n = 34	P value A vs B	RSV- A/NA1 n = 62	RSV- A/ON1 n = 69	P value A/NA1 vs A/ON1
	n/N (%)	n/N (%)		n/N (%)	n/N (%)	
**Demographic and clinical presentation**						
Males (%)	79 (60.3)	22 (64.7)	0.78	35 (56.5)	44 (63.8)	0.49
Mean age ± SD, yrs	0.87 ± 0.81	0.97 ± 1.06	0.82	0.88 ± 0.65	0.86 ± 0.93	0.21
Presence of fever” (%)	126 (96.2)	30 (88.20)	0.08	59 (95.2)	67 (97.1)	0.66
High-grade fever° (%)	83 (63.3)	15 (44.1)	0.06	45 (72.6)	38 (55.1)	0.05
Respiratory rate, bpm	50.0 ± 13.4	42.2 ± 9.9	0.43	52.3 ± 9.3	47.9 ± 16.5	0.52
Mean SpO_2_ in room air ± SD, %	95.9 ± 2.8	97.3 ± 2.7	0.88	96.4 ± 2.5	95.0 ± 3.0	0.79
Diagnosis						
Upper respiratory tract infection	59 (45.0)	15 (44.1)	0.92	10 (16.1)	49 (71.0)	**<0.0001**
Lower respiratory tract infection	72 (55.0)	19 (55.9)		52 (83.9)	20 (29.0)	
**Clinical outcome**						
Hospitalisation, No.(%)	45 (34.3)	16 (47.0)	0.24	29 (46.8)	16 (23.2)	**0.007**
Mean duration of hospitalisation, days ± SD	5.7 ± 2.5	6.8 ± 1.8	0.06	5.6 ± 2.7	6.0 ± 2.1	0.32
Drug use, No. (%)						
Antibiotics	126 (96.1)	34 (100.0)	0.58	59 (95.2)	67 (97.1)	0.66
Antipyretics	126 (89.0)	30 (88.2)	0.08	61 (98.4)	65 (94.2)	0.36
Aerosol therapy	120 (91.6)	27 (82.3)	0.06	57 (91.9)	63 (91.3)	0.85
Mean absence from community, days ± SD	10.1 ± 4.2	16.5 ± 12.0	0.08	10.9 ± 4.7	8.2 ± 2.0	0.44
Similar illness within the family	62 (47.3)	19 (55.9)	0.48	27/62 (43.5)	13/23 (56.5)	0.29
**Laboratory data**						
White blood cell count, cells/μL	11453 ± 4361	9208 ± 2511	0.05	11248 ± 4840	11997 ± 2770	0.29
Neutrophils, %	42.9 ± 17.2	46.5 ± 13.0	0.40	39.4 ± 16.0	51.8 ± 17.4	**0.02**
Lymphocytes, %	43.3 ± 14.5	40.9 ± 12.2	0.71	44.4 ± 14.0	21.7 ± 5.2	**0.03**
Monocytes, %	13.6 ± 4.8	12.6 ± 3.9	0.65	13.9 ± 4.8	8.7 ± 2.3	**0.04**
Basophils, %	0.4 ± 0.3	0.5 ± 0.7	0.90	0.4 ± 0.4	0.5 ± 0.2	0.71
Eosinophils, %	0.4 ± 0.5	0.5 ± 0.4	0.64	0.4 ± 0.5	0.1 ± 0.1	0.76
CRP, μg/dL	16.9 ± 80.8	2.6 ± 3.8	0.56	3.4 ± 4.7	47.6 ± 144.1	0.48

bpm, beats per minute; CRP, C-reactive protein; SD, standard deviation; SpO_2_, peripheral oxygen saturation.”≥38.0°C any time during illness (before or upon enrolment, or during follow-up); ≥39.0°C any time during illness (before or upon enrolment, or during follow-up).

However, comparison of the children infected by genotypes A/NA1 and A/ON1 showed that LRTI was more frequently diagnosed in those with A/NA1 infection (p<0.0001), who were also more frequently hospitalised (p = 0.007); moreover, their white blood cell counts included a significantly higher number of lymphocytes (p = 0.03) and monocytes (p = 0.04). On the contrary, there was no difference in the clinical characteristics of the children infected by the three phylogenetic subgroups in the A/ON1 strain, or between those of the children with B/BA9 or B/BA10 infection (data not shown). However, the infections due to B strains were globally more severe than those due to A/ON1 because the children with B viruses were more frequently affected by LRTIs and more frequently hospitalised (Table 3).

## Discussion

The findings of this study show that RSV has complex circulation patterns that are characterised by the periodic replacement of the predominant genotypes with new ones during successive epidemic seasons. As previously found in other countries [13,17–21] and Central Italy [16], the new RSV-A/ON1 strain emerged in Northern Italy in 2011–2012, and soon completely replaced the previously dominant RSV-A/NA1 strain. In addition, as recently occurred in Japan [10], RSV-B/BA9 was predominant between RSV-B viruses. It was identified in all the years of the study, whereas detection of RSV-B/BA10 was limited to 2010–2011 and 2012–2013 seasons. Phylogenetic and deduced amino acid sequence analyses of the RSV strains revealed that ongoing changes characterised the different clusters in the RSV-A tree [31,32]. Interestingly, almost all of the Italian NA1 strains lost the first N-glycosylation site due to substitution N237D [5], whereas this N-glycosylation site was restored in three NA1 and all ON1 strains. The NA1 strains fell into two clusters characterised by a number of amino acid changes, but it is not clear how these changes favoured the evasion of the host immune response. The ON1 genotype was characterised by E232G, T253K and P292L substitutions, as previously reported in Chinese strains [33], and other cluster-specific mutations were observed in ON1 clusters A (L274P, L298P and 310P) and A1 (G254E, Y273H, and Y297H) [18,34].

It has been suggested that the evolution of the major antigens of RSV and other respiratory viruses is associated with selective pressure due to host immune responses [35]. Our search for phylogenetic signatures of selective pressure in RSV-A and RVS-B showed that the RSV genes have predominantly negatively or neutrally selected evolving sites. Only a minority were under positive selection, thus suggesting that the forces driving the evolution of RSV are mainly stochastic. However, additional investigations are needed to evaluate whether amino acid changes in the G protein may help the virus to alter the antigenic characteristics leading to an evolutionary advantage.

The estimated overall mean *dN/dS* ratios of both RSV-A and RSV-B were in line with those reported by other authors [33,36]. Substitutions at positions 274 and 286 (corresponding to 310 in our RSV-A alignment) have previously been associated with antibody escape [35,37]. The IFEL method indicated that the RSV-A and RSV-B sequences had respectively four and two codons under positive selection pressure and, in agreement with other reports [38], this finding suggests a variation within the lineage corresponding to emerging RSV genotypes.

In this study, only children exclusively infected by RSV were included. This permitted to have precise information regarding the real pathogenic role of this virus avoiding the risk of misinformation that can occur when all the RSV infections, including co-infections between RSV and other respiratory viruses, are studied. However, only children aged < 2 years attending the Emergency Room were enrolled. Moreover, in comparison to RSV-B, a significant greater number of RSV-A positive cases was identified. These are limits that do not allow to draw firm conclusions about the real global clinical relevance of the different RSV types in paediatrics. RSV infection is more severe in younger children and mild to moderate infections are usually followed by primary care paediatricians in the community [1]. However, in the study population, we did not find any difference in the severity of the disease due to RSV-A and RSV-B strains. This finding is in line those of some previous studies [39–41], but conflicts with those of other epidemiological evaluations showing a significantly higher risk of severe disease due to type A [42–45] or type B [46]. Moreover, it is not agree with findings in various cell culture and *in vivo* models suggesting that individual RSV-A strains may be more infective, virulent and immunopathogenic [1]. In this regard, it is interesting to note the findings of a recent *in vitro* study of primary epithelial cells and epithelial cell lines, which suggest that prototypical RSV-A and RSV-B strains are differently capable of inducing nuclear factor-κB (NF-κB) activation (i.e. an important step in the cascade of events leading to the production of pro-inflammatory cytokines) and the subsequent induction of the NF-κB responsive genes interleukin(IL)-6 and IL-8, with RSV-B eliciting significantly weaker responses than RSV-A [47].

These conflicting results may be due to differences in study design, disease definition, inclusion criteria, or genotyping methodology, but they may also be at least partially explained by a real difference in the pathogenic role of some particular genotypes. The greater severity of the infections due to RSV-A/NA1 in comparison with RSV-A/ON1 suggest that different RSV genotypes may be associated with different degrees of virulence, and that the relevance of one RSV type in clinical practice may depend on the incidence of infections due to the most virulent genotype during the period of the epidemiological evaluation. A simple evaluation of RSV type is not enough to predict the risk of severe infection during epidemics, and only studies specifically planned to evaluate the virulence of the different genotypes can allow a precise assessment of what can be expected when a particular genotype is in circulation.

We found that RSV-A/ON1 was significantly less virulent than genotype A/NA1, as shown by the lower incidence of LRTIs and less frequent hospitalisation of A/ON1-infected children. This is in line with the findings of other authors [16,17,31,48], although Prifert *et al*. observed that a considerable number of children with A/ON1 infection were admitted to an intensive care unit [31], possibly because they only enrolled children with LRTIs. The lower virulence of A/ON1 also seems to be further supported by our finding that the clinical manifestations due to A/ON1 infection were significantly less severe than those due to the simultaneously circulating B/BA9 and B/BA10 viruses. Similar data have been reported by Panayiotou *et al*. [48], who showed that patients infected with RSV-A/ON1 had a significantly lower mean severity score than children with B/BA infection.

In conclusion, the findings of this study highlight the value of investigating the circulation of RSV strains and evaluating the different pathogenic roles of the various genotypes during mild and severe RSV infection because each individual strain can have a different impact on the host. A knowledge of the correlations between types, genotypes and disease severity may also be important in order to be able to include the more virulent strains in future vaccines.
